# An overview on disulfide-catalyzed and -cocatalyzed photoreactions

**DOI:** 10.3762/bjoc.16.118

**Published:** 2020-06-23

**Authors:** Yeersen Patehebieke

**Affiliations:** 1 School of Chemistry and Chemical Engineering, Nanjing University, Nanjing, 210023, China

**Keywords:** cycloaddition, disulfide catalyst, isomerization, oxidation, photocatalysis, thiyl radical

## Abstract

Disulfides are versatile catalysts. They can be photocatalysts, hydrogen atom transfer (HAT) catalysts, cocatalysts, or initiators in photocatalytic reactions. Under photoirradiation, organic disulfides can be easily cleaved into free thiyl radicals (RS^•^) and can reversibly add to unsaturated multiple bonds to catalyze a variety of functionalization reactions under mild conditions. In photoredox catalysis reactions, an excellent electron transfer ability and excellent radical properties also made these thiyl radicals powerful HAT catalysts. They have increasingly been proven useful in various types of organic photoreactions, such as cyclizations, anti-Markovnikov additions, aromatic olefin carbonylations, isomerizations, etc. They are a class of green, economic, mild, and chemoselective radical catalysts that deserve more attention. The present review highlights the recent progress in the field of disulfide-catalyzed and -cocatalyzed photocatalytic reactions for different reaction types.

## Introduction

Organic disulfides are often used as the skeleton for drugs, pesticides, rubber auxiliaries, polymers, and electronic materials [1]. Over the past decade, organic disulfide-involving photoreactions have attracted increasing attention. Disulfides have versatile catalytic abilities: they can be a photocatalyst, HAT catalyst, initiator, or cocatalyst in organic synthesis. The thiyl radicals (RS^•^) formed under illumination conditions have the unique ability of promote radical bond-forming reactions. Their ability to reversibly add to unsaturated bonds, promoting a variety of reactions, renders them a class of green, economic, mild, and chemoselective radical catalyst. Apart from this, they are also excellent HAT catalysts in photoredox catalysis systems [2–3]. In various types of organic photochemistry reactions, such as cyclizations, anti-Markovnikov additions, oxidations, or isomerizations, disulfides have increasingly proven their power. Herein, we briefly describe the progress in the field of disulfide-involving photocatalysis in recent years for different reaction types.

## Review

### Cycloaddition reactions

**Scheme 1:**
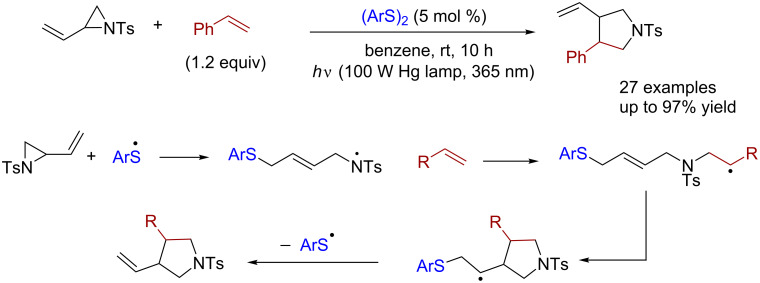
[3 + 2] cyclization catalyzed by diaryl disulfide.

As early as 1988, Feldman and co-workers reported an example of a [3 + 2] cycloaddition reactions under UV irradiation with azobis(isobutyronitrile) (AIBN) as the free radical initiator and phenyl disulfide as the catalyst, in which the three-membered rings containing double bonds and substituted olefins were transformed into five-membered-ring structures with high regioselectivity [4]. Based on this reaction, Maruoka reported in 2016 that *N*-tosylvinylaziridines and alkenes could undergo cyclization reactions to generate pyrrolidines, catalyzed by substituted aryl disulfides under ultraviolet-light irradiation (Scheme 1) [5].

**Scheme 2:**
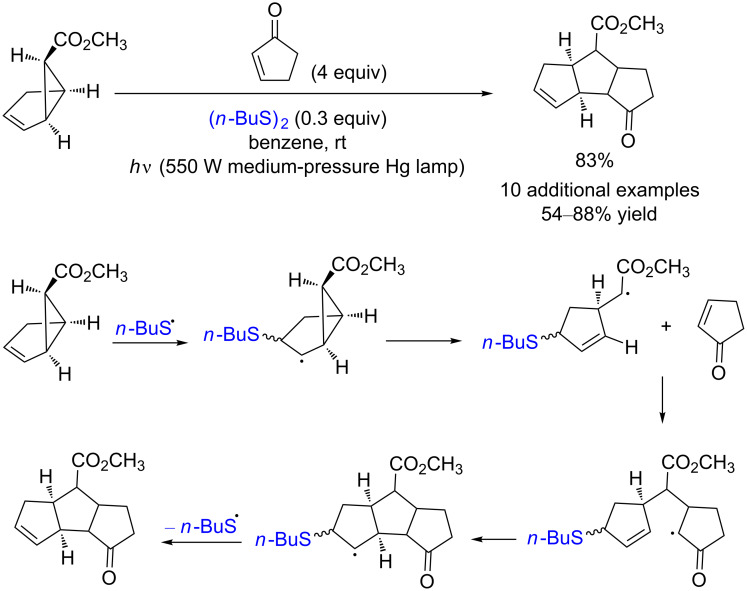
[3 + 2] cycloaddition catalyzed by disulfide.

In 1997, Jung and co-workers used similar disulfide-catalyzed [3 + 2] cycloaddition reactions for the synthesis of polycyclic frameworks. The irradiation of vinylcyclopropanes with alkenes or alkynes in the presence of dibutyl disulﬁde afford the desired bi- or tricyclic products with 54–88% yield (Scheme 2) [6].

**Scheme 3:**
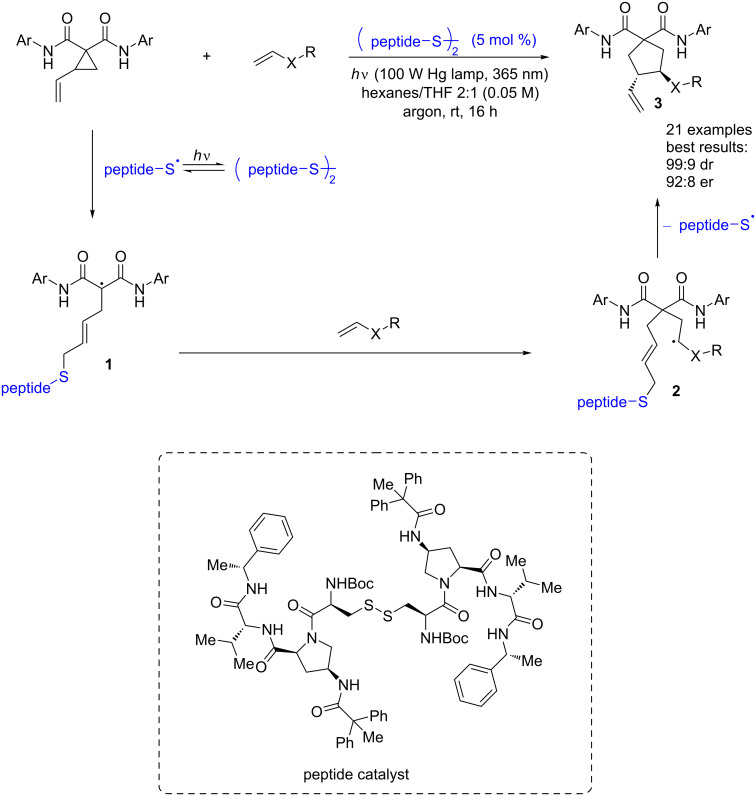
Disulfide-bridged peptide-catalyzed enantioselective cycloaddition.

In 2014, Maruoka and co-workers reported an excellent thiyl radical-catalyzed enantioselective cyclization reaction of vinylcyclopropanes with alkenes [7]. For the extension of this concept, in 2018, Miller and co-workers reported a UV-light-promoted disulfide-bridged peptide-catalyzed enantioselective cycloaddition of vinylcyclopropanes with olefins [8]. The reaction mechanism of this cycloaddition process was similar to other thiyl radical-catalyzed cycloaddition cascade reactions. The alkylthiyl radical generated by the homolysis of a disulfide-bridged peptide precatalyst under UV-light irradiation triggers the reaction by adding to vinylcyclopropane to form the radical intermediate **1**. Then, the addition of olefins to this intermediate **1** gives the intermediate **2**, and the succeeding cyclization step yields the final product **3** and regenerates the active thiyl radical catalyst (Scheme 3). Vinylcyclopropanes engage with the peptide backbone via an H-bonding interaction, and in order to achieve a high enantioselectivity, the amide functionalization of the peptide at the 4-proline position is essential. Amide-substituted vinylcyclopropanes have a relatively good H-bond-donating ability, so they are more successful substrates for this transformation. Although multiple olefins can be successful olefinic coupling partners for this reaction, electron-rich olefins bearing an α-heteroatom would be preferable for achieving a better enantioselectivity.

**Scheme 4:**
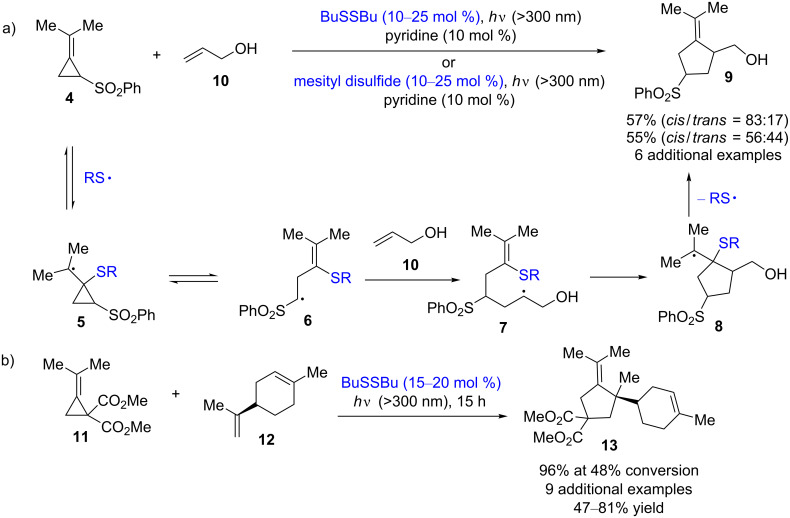
Disulfide-catalyzed [3 + 2] methylenecyclopentane annulations.

In 1990, Singleton and co-workers reported disulfide-catalyzed [3 + 2] methylenecyclopentane annulations of olefins with methylenecyclopropanes. This regioselective, mild, and protecting group-free annulation requires only an equimolar amount of the reacting alkene and does not require an excess of the reacting alkene, unlike other methods [9]. Furthermore, the stereochemistry of the product can be modulated by changing the stereochemical structure of the disulfide catalyst [10]. The simple reaction mechanism is shown in Scheme 4a. Thiyl radicals, which are generated under irradiation, trigger the reaction by adding to the methylenecyclopropane **4**, which affords the cyclopropylcarbinyl radical **5**. Then, the radical **5** opens to form the homoallylic radical **6**, followed by the addition of olefins to this radical form the 5-hexenyl radical **7**. The ring closure of the radical **7** and the elimination of a thiyl radical furnishes the final product **9**. When it came to annulations of complex olefins, this method could not give satisfactory results. In order to overcome this weakness, the authors also developed disulfide-catalyzed [3 + 2] methylenecyclopentane annulations of unactivated alkenes with methylenecyclopropanecarboxylates and -dicarboxylates **11** (Scheme 4b) [11]. This annulation method is also applicable for the synthesis of a wide variety of cyclopentane derivatives [12].

**Scheme 5:**
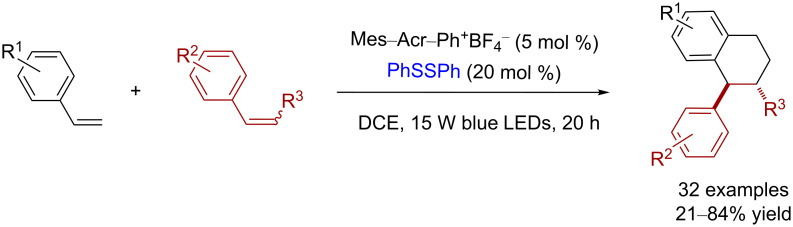
Disulfide as a HAT cocatalyst in the [4 + 2] cycloaddition reaction.

In 2017, Huang and co-workers reported a [4 + 2] cycloaddition reaction promoted by blue LED light, using aromatic olefins as the precursor, an acridinium photoredox catalyst (Mes–Acr–Ph^+^BF_4_^−^), and disulfide as the HAT cocatalyst, to generate the tetralin skeleton, which is widely seen in drugs and pesticide synthesis (Scheme 5) [13]. Diphenyl disulfide played an important role in the [4 + 2] cycloaddition process. Without diphenyl disulfide, only the product of the [2 + 2] cycloaddition was observed.

**Scheme 6:**
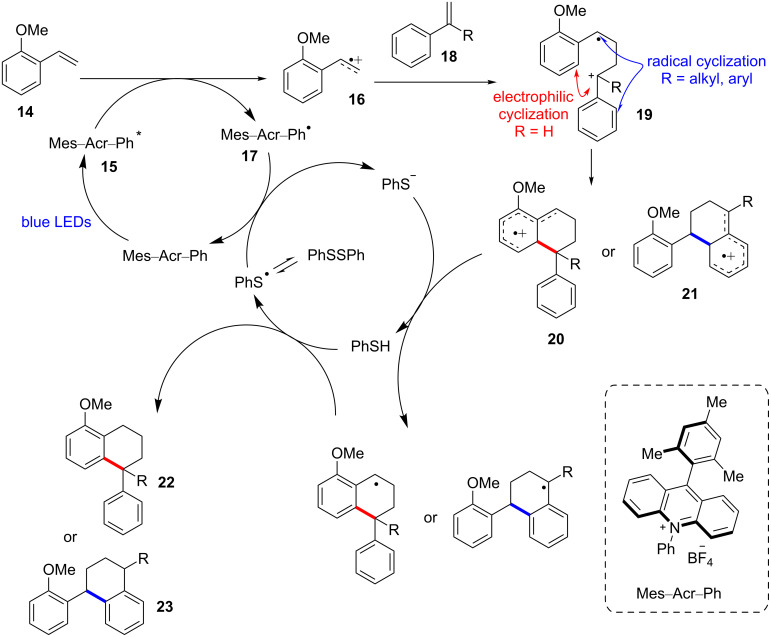
Proposed mechanism of the [4 + 2] cycloaddition reaction using disulfide as a HAT cocatalyst.

Huang and co-workers proposed a polar radical crossover cycloaddition mechanism for this Diels–Alder cycloaddition (Scheme 6). The electron transfer from the electron-rich styrene **14** to the activated acridinium photocatalyst **15** oxidizes the styrene **14** to form the styrene radical **16** and the acridine radical **17** (Mes–Acr–Ph^•^). The subsequent reaction of the formed styrene radical **16** with another styrene **18** gives the radical species **19** and the reoxidation of the acridine radical **17** by a thiyl radical, which is generated by the homolysis of diphenyl disulfide, regenerating the photocatalyst. In a nonpolar solvent, PhSSPh accelerates the [4 + 2] cycloaddition of the radical cation **19**, but the electron-relay catalyst promotes the [2 + 2] cycloaddition. The radical cation **19** can undergo two different types of cyclizations, subject to the relative reactivity of its radical and cation center. The α-substituted cation **19** favors the radical cyclization path because the stabilization by an α*-*substituent makes its cation center more stable and less reactive. In contrast, the non-α*-*substituted cation **19** prefers the cationic cyclization. However, the two cyclization processes form the intermediates **20** and **21**, respectively, which have the same 5-electron 6-carbon cation radical character. Finally, the subsequent deprotonation and HAT by PhS^−^ and PhSH yields the desired [4 + 2] cycloaddition products **22** and **23**, respectively.

**Scheme 7:**
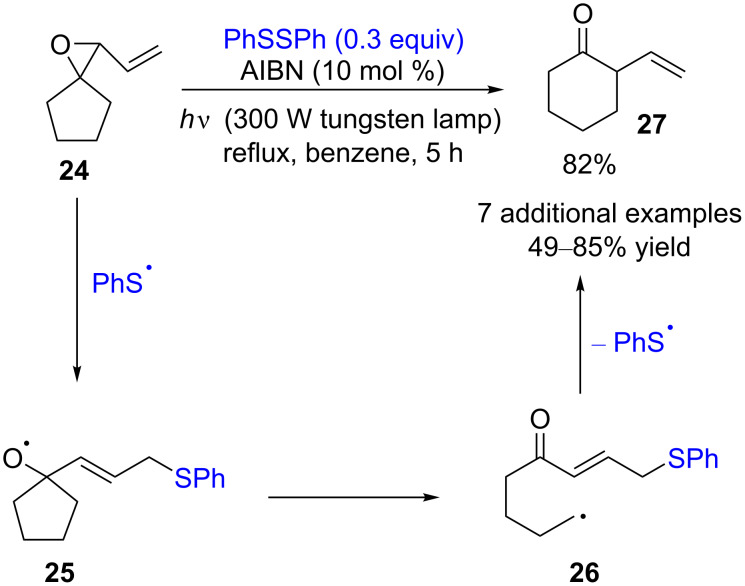
Disulfide-catalyzed ring expansion of vinyl spiro epoxides.

In 1991, Kim and co-workers reported a disulfide-catalyzed ring expansion of cyclobutanone, cyclopentanone, and three carbon ring-expanded 1,3-diones from vinyl spiro epoxides [14]. The reaction is initiated by the addition of a thiyl radical to the vinyl epoxide **24**, followed by the epoxide fragmentation to the alkoxy radical **25**. Then, the β-cleavage to form the carbon-centered radical **26**, the final cyclization, and an elimination give the desired product **27** (Scheme 7). The use of PhSSPh obviated the undesired reactions, which occurred when using Ph_3_SnH and Bu_3_SnH, respectively, as the catalyst for this reaction.

### Oxidation reactions

**Scheme 8:**
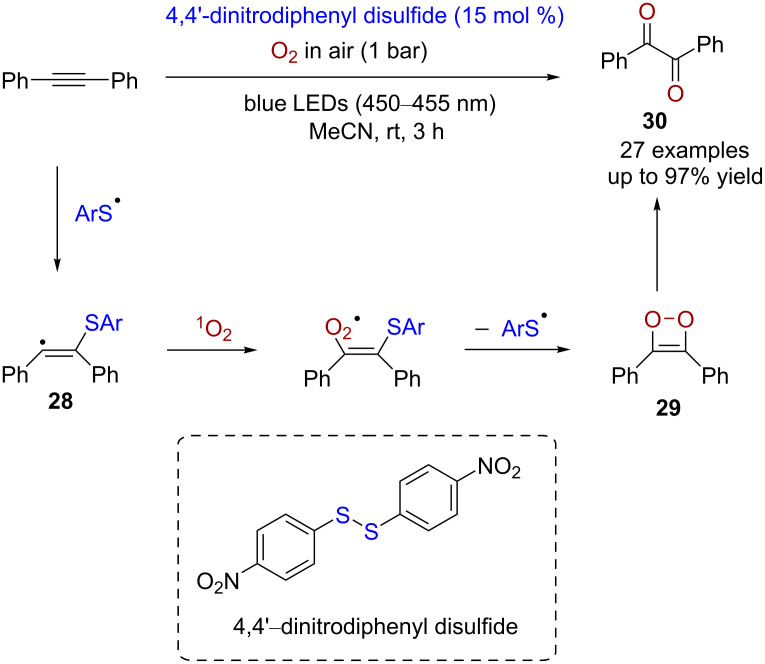
Disulfide-catalyzed aerobic oxidation of diarylacetylene.

Not only can disulfide catalysts induce cycloadditions of olefins but also catalyze the formation of four-membered ring intermediates between double or triple bonds and oxygen, and thus converting unsaturated hydrocarbons to carbonyl compounds. Regarding the oxidation of alkynes, Wang reported a method for preparing diaryl-1,2-diketones from diarylalkynes in the presence of visible light, oxygen, and disulfide [15]. The diaryl-1,2-diketone products can serve as an important structural component of many natural products and bioactive molecules. This method works for a broad range of substrates with high yields (77–97%). A plausible mechanism for this reaction is that by the excitation with visible light, the homolytic cleavage of disulfide generates an arylthiyl radical (ArS^•^), which adds to diphenylacetylene to form a free-radical intermediate **28**. Subsequently, this intermediate traps a molecule of singlet oxygen (^1^O_2_), and the thiyl radical is regenerated to give the four-membered ring intermediate **29**. Finally, the rearrangement of the four-membered intermediate provides the diketone **30** as the product (Scheme 8).

**Scheme 9:**
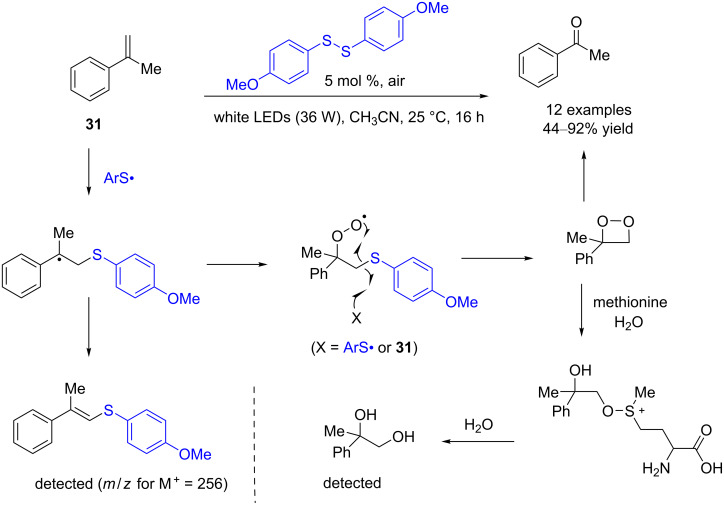
Disulfide-catalyzed aerobic photooxidative cleavage of olefins.

In 2017, Wang and co-workers reported an oxidative cleavage of aromatic alkenes at ambient temperature with visible-light irradiation, using electron-rich aryl disulfides as the photocatalyst and oxygen as the oxidant [16]. At room temperature, bis(4-methoxyphenyl) disulfide was employed as the metal-free photocatalyst under visible-light and 1 bar of O_2_ to realize the aerobic oxidative cleavage of the C=C bonds. Under these mild conditions, monosubstituted and 1,1-disubstituted arylolefins could be effectively cleaved into the corresponding aldehydes or ketones. The proposed mechanism is that disulfide is split by visible light to form thiyl radicals, which catalyzes the combination of the olefin with O_2_ to form the intermediate dioxetane that decomposes into ketone or aldehyde products (Scheme 9). However, in the absence of light or oxygen, disulfide could not catalyze the oxidative cleavage of olefins. It was proposed that disulfide and the olefin might be able to form a charge-transfer complex, which may rationalize the unconventional homolysis of the aromatic S–S bond by visible light.

**Scheme 10:**
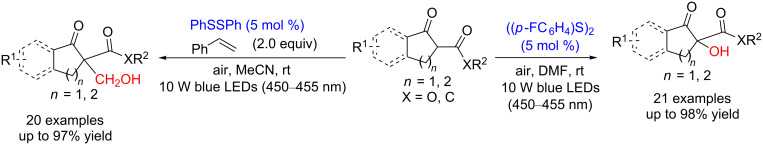
Disulfide-catalyzed aerobic oxidation of 1,3-dicarbonyl compounds.

In 2019, Meng and co-workers reported a visible light-mediated disulfide-catalyzed metal-free and base-free α*-*functionalization of 1,3-dicarbonyl compounds [17]. Under visible-light irradiation, the α*-*hydroxylation or α*-*hydroxymethylation of 1,3-dicarbonyl compounds, was efficiently implemented via this disulfide, which induced an aerobic oxidation. The hydroxylation and hydroxymethylation of a broad range of β-keto esters and β-keto amides that had electron-donating or -withdrawing groups on the phenyl ring gave good to excellent yields (42–98%, Scheme 10). One exceptional decrease in the yield of the hydroxylation product (13–33%) occurred when β-keto esters with methoxy groups on the phenyl ring were used, but the hydroxymethylation yield was just undulated slightly. Other carbonyl compounds, 1,3-diones, and functionalized five-, six-, and seven-membered keto ester derivatives did not show any desired reaction. In combination with a continuous-flow strategy, this disulfide-catalyzed aerobic oxidation process can be scaled up to a gram-scale.

**Scheme 11:**
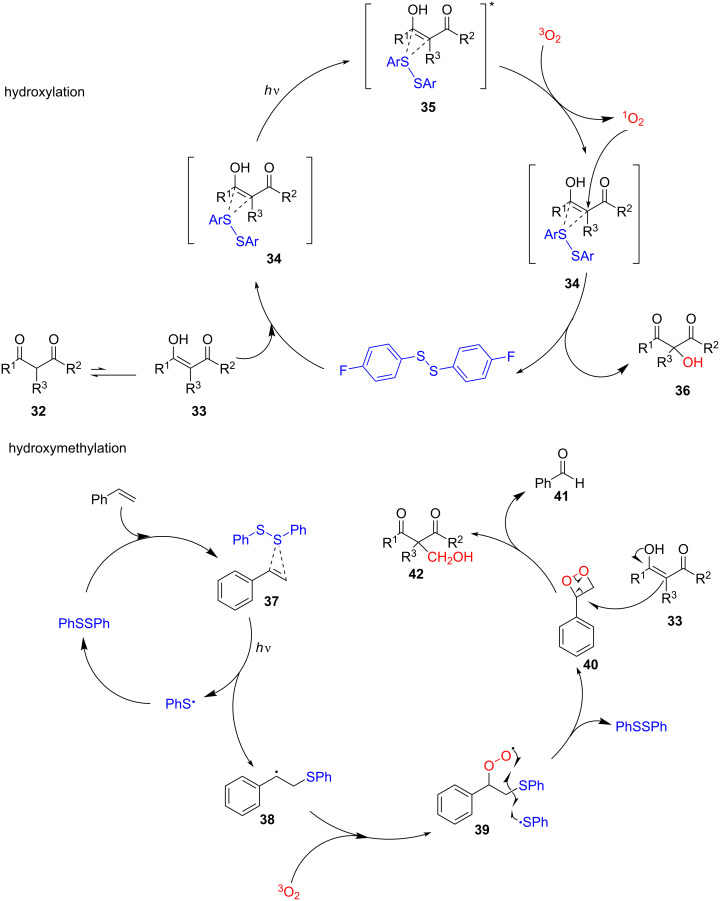
Proposed mechanism of the disulﬁde-catalyzed aerobic oxidation of 1,3-dicarbonyl compounds.

A plausible mechanism of the reaction is shown in Scheme 11. The hydroxylation starts with the formation of the photoexcited disulfide–enol complex **35** from the disulfide–enol complex **34** under irradiation. Then, the photoexcited complex **35** reacts with triplet oxygen (^3^O_2_) to yield the reactive ^1^O_2_. The subsequent reaction of singlet oxygen with the disulfide–enol complex **34** furnishes the desired hydroxylation product **36** and frees the disulfide for the next cycle. The generation of the disulfide–olefin complex **37** from styrene and disulfide initiates the hydroxymethylation. The following ^3^O_2_ trapping by the intermediate **38**, which is generated by the addition of a thiyl radical to the styrene under irradiation, gives the intermediate species **39**. The abstraction of a thiyl radical from the intermediate **39** yields the key intermediate dioxetane **40** and regenerates the disulfide. Finally, the reaction between the enol **33** and the dioxetane **40** affords benzaldehyde (**41**) and the product **42**.

**Scheme 12:**
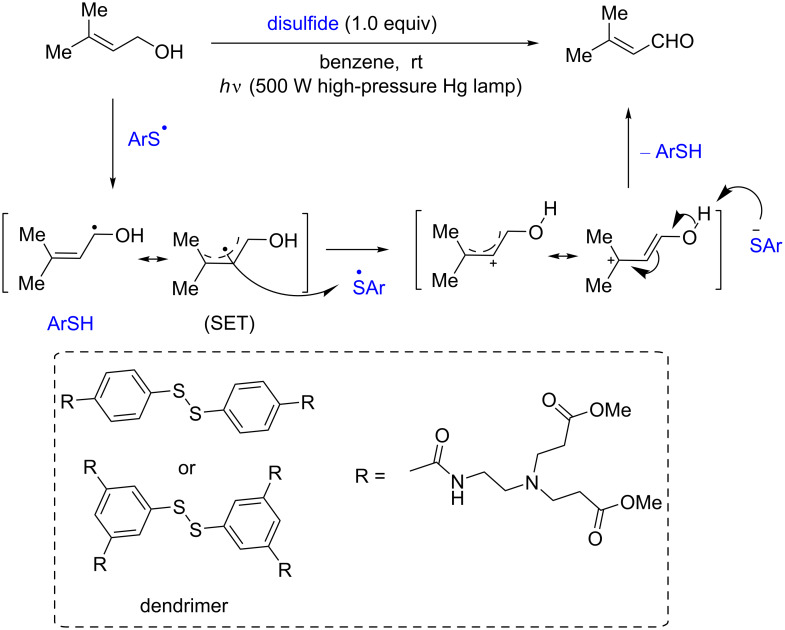
Disulfide-catalyzed oxidation of allyl alcohols.

In 2008, Tsuboi and co-workers reported a photoinduced oxidation of allyl alcohols to acrylaldehydes at room temperature, catalyzed by diphenyl disulﬁde derivatives [18]. In the proposed mechanism, disulfide is split by the photoirradiation to form thiyl radicals. Then, the abstraction of the α*-*hydrogen atom from allyl alcohol by the thiyl radical produces thiophenol and an allylic radical. Next, the single-electron transfer (SET) from the allylic radical to another thiyl radical generates the allylic cation. Subsequently, the proton abstraction from the hydroxy group by the SET-generated thiolate gives the final oxidation product (Scheme 12). Interestingly, compared to the diphenyl disulﬁde-catalyzed oxidation, which gives only 23–44% yield, the use of dendrimer disulﬁdes gives the products in much better yields (38–63%).

**Scheme 13:**
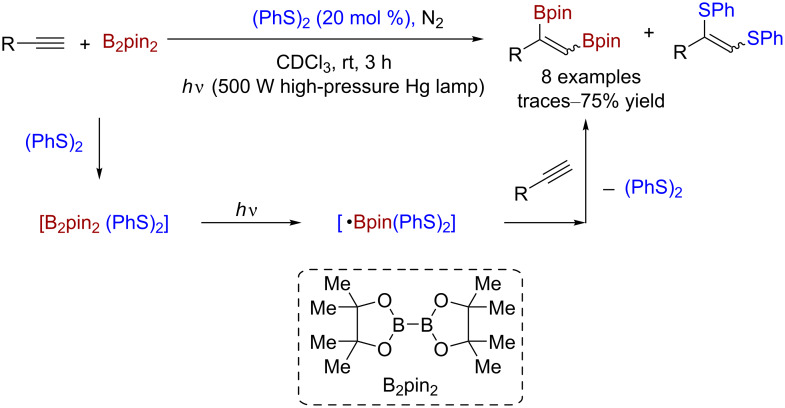
Disulfide-catalyzed diboration of alkynes.

Similar to oxidations, disulfide can also be used as the catalyst to construct functional groups that contain heteroatoms. Ogawa and co-workers reported a class of efficient diboration reactions. Under light irradiation, disulfide was used as the photocatalyst to facilitate the addition of bis(pinacolato)diboron (B_2_pin_2_) to terminal alkynes [19], and the corresponding diboryl alkenes were generated with a yield of 43–75% (Scheme 13).

### Reductive dehalogenation reactions

**Scheme 14:**
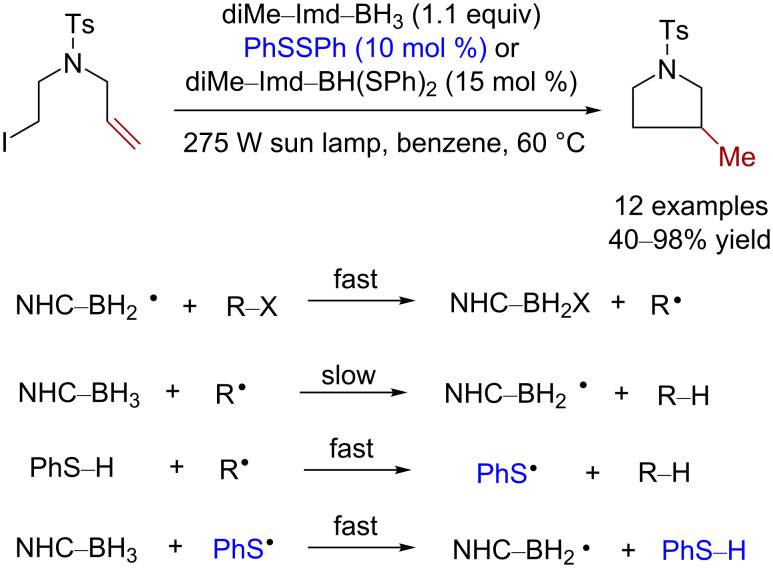
Dehalogenative radical cyclization catalyzed by disulfide.

In 2013, Curran and co-workers reported a light-induced dehalogenative reduction with polarity reversion catalyzed by diphenyl disulfide or boryl bissulfide (diMe–Imd–BH(SPh)_2_) as the catalyst and 1,3-dimethylimidazol-2-ylidene·borane (diMe–Imd–BH_3_) as the initiator [20]. Thiyl radicals formed by diphenyl disulfide under light can further generate the active HAT catalyst thiophenol in situ. The yield of the reductive dehalogenation or dehalogenative radical cyclization could reach up to 40–98% (Scheme 14).

**Scheme 15:**
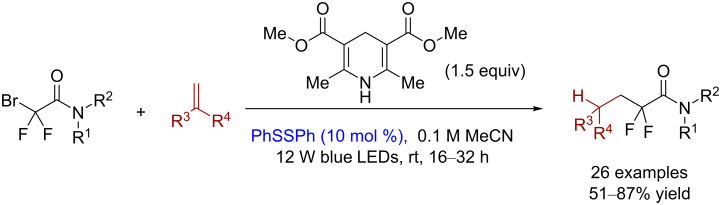
Hydrodiﬂuoroacetamidation of alkenes catalyzed by disulfide.

The introduction of fluorine-containing groups can effectively change the physical and chemical properties and biological activities of organic molecules [21–22]. The addition reaction using disulfide as the visible-light catalyst is an effective and atom-economic method for introducing fluorine-containing groups. In 2016, Cheng and co-workers reported a hydrodiﬂuoroacetamidation of alkenes in which disulfide served as the photocatalyst with the Hantzsch ester as the reducing agent (Scheme 15) [23]. These reactions do not require costly transition-metal complexes, and they do not involve the oxidative regeneration of a photoredox catalyst or a carbocation species, which suppresses the formation of side products that are observed when using other methods.

**Scheme 16:**
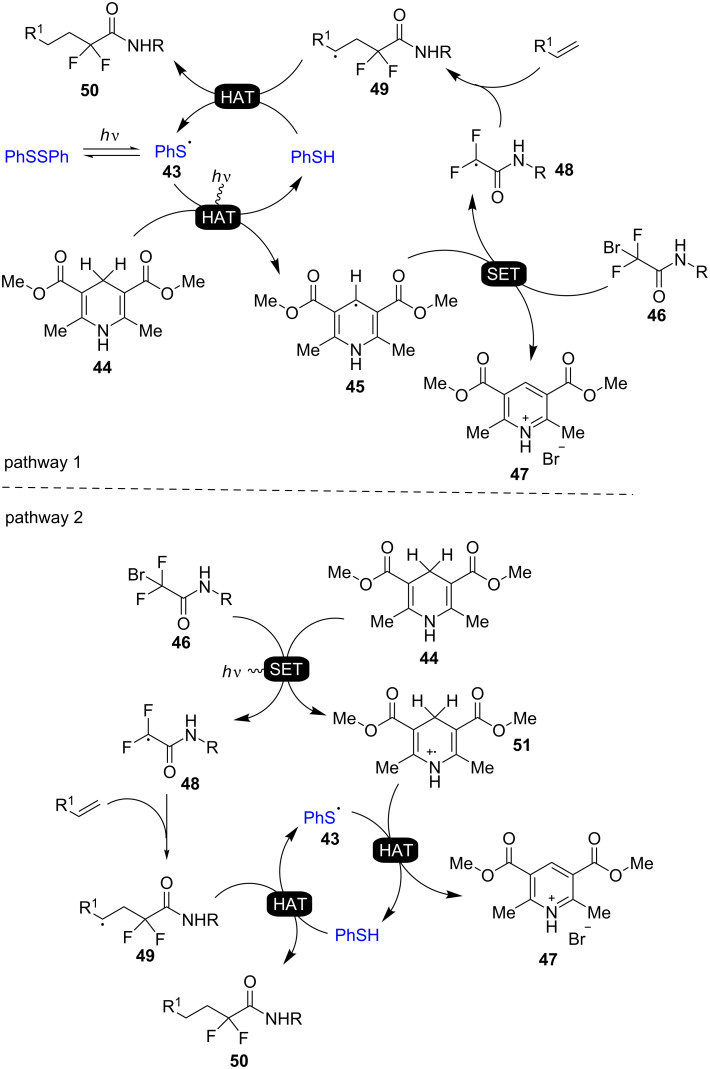
Plausible mechanism of the hydrodiﬂuoroacetamidation of alkenes catalyzed by disulfide.

The suggested mechanism by Cheng and co-workers is shown in Scheme 16. The reaction could go through two possible pathways. The generation of the phenyl thiyl radical **43** by homolysis of diphenyl disulfide under visible light triggers the HAT-initialized pathway 1. The hydrogen abstraction from the Hantzsch ester **44** by the thiyl radical **43** gives the ester radical **45**. Then, the subsequent SET from the Hantzsch ester radical **45** to the acetamide **46** forms the amide radical **48**, the protonated pyridine **47**, and bromide. The following addition of alkenes to the generated difluoroamide radical **48** gives the intermediate **49**. Finally, the desired difluoroalkylation product **50** is produced by a subsequent HAT from thiophenol to the intermediate **49**, and the thiyl radical **43** regenerates for the next catalytic cycle. The other plausible reaction pathway 2 starts with a SET from the excited Hantzsch ester **44** to the acetamide **46** to form the intermediate difluoroamide radical **48** and the cationic radical **51**. The subsequent addition of alkenes to the formed radical **48** and HAT from the intermediate **51** to the phenyl thiyl radical **43** yields the intermediate **49**, thiophenol, the protonated pyridine **47**, and bromide. Finally, HAT from the thiophenol to the intermediate **49** gives the difluoroalkylation product **50**.

### Alkene functionalization reactions

**Scheme 17:**
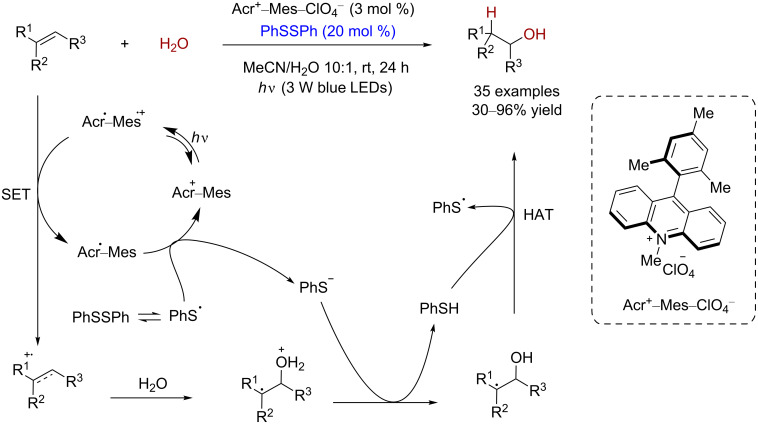
Disulfide-cocatalyzed anti-Markovnikov olefin hydration reactions.

The olefin hydration is an important method to synthesize alcohols, and realizing the anti-Markovnikov regioselectivity is extremely challenging. In 2017, Lei and co-workers reported a class of disulfide-cocatalyzed anti-Markovnikov olefin hydration reactions [24]. In this reaction, 9-mesityl-10-methylacridinium (Acr^+^–Mes–ClO_4_^−^) and diphenyl disulfide are used as the photoredox catalyst to prepare the corresponding primary and secondary alcohols from terminal and internal oleﬁns. The substrate scope is broad, with excellent regioselectivities and yields up to 96% (Scheme 17).

### Decarboxylation reactions

**Scheme 18:**
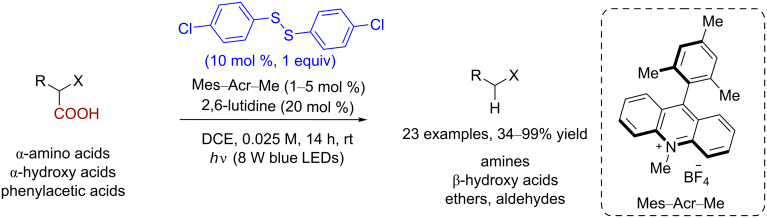
Disulfide-catalyzed decarboxylation of carboxylic acids.

Carboxylic acid often serves as an inexpensive and abundant source of biomass-derived molecules. Decarboxylative transformations of carboxylic acids into value-added chemical products (such as biofuels) are a key objective in organic synthesis [25]. In 2014, Wallentin and co-workers reported a type of decarboxylation reaction of α*-*amino acids, α*-*hydroxy acids, and phenylacetic acids using an acridinium photoredox catalyst (Mes–Acr–Me) and bis(4-chlorophenyl) disulfide as catalysts (Scheme 18) [26].

**Scheme 19:**
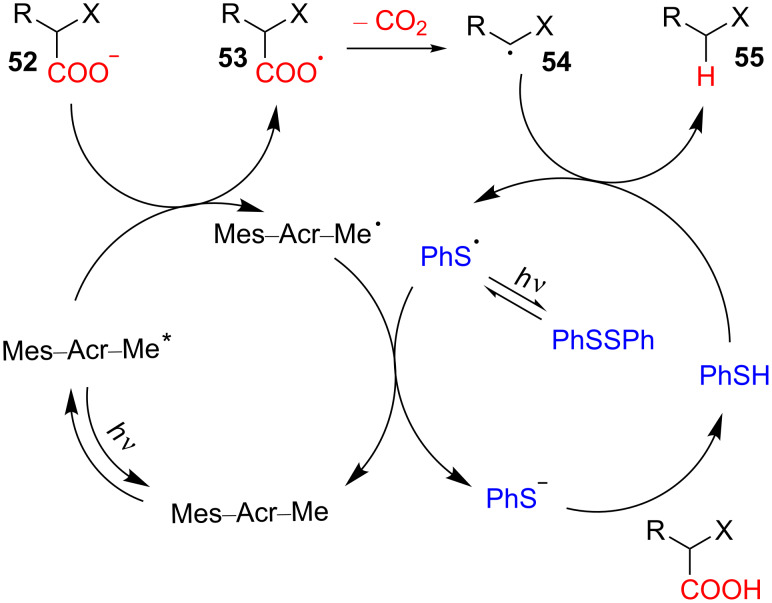
Proposed mechanism of the disulfide-catalyzed decarboxylation of carboxylic acids.

The proposed mechanism for this reaction is shown in Scheme 19. The reaction begins with the photoexcitation of the catalyst Mes–Acr–Me, which generates the active species Mes–Acr–Me*. Then, the single-electron oxidation of the deprotonated carboxylic acid **52** with Mes–Acr–Me* results in the formation of an acyloxyl radical **53**. The subsequent rearrangement of the radical **53** gives a carbon-centered radical **54** and carbon dioxide. The phenyl thiyl radical formed via the illumination of disulfide reoxidizes the reduced catalyst to regenerate the ground state of the photocatalyst Mes–Acr–Me. Then, the thiolate anion is protonated to give thiophenol, which then acts as a hydrogen-atom donor towards the carbon-centered radical **54** to furnish the final hydrodecarboxylation product **55**.

**Scheme 20:**
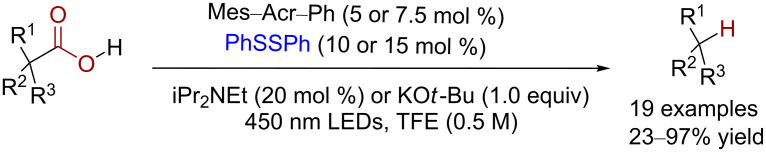
Disulfide-catalyzed decarboxylation of carboxylic acids.

Nicewicz and co-workers also reported the decarboxylation of carboxylic acids and malonic acid derivatives catalyzed by an acridinium photoredox catalyst (Mes–Acr–Ph) and PhSSPh in 2015 (Scheme 20) [27]. This direct method of the organocatalytic decarboxylation of carboxylic acids to alkanes provided an efficient protocol for the synthesis of alkanes from carboxylic acid substrates previously inaccessible through other methods. The mechanism of this reaction is similar to the previously proposed mechanism in Scheme 19.

### Isomerization reactions

**Scheme 21:**
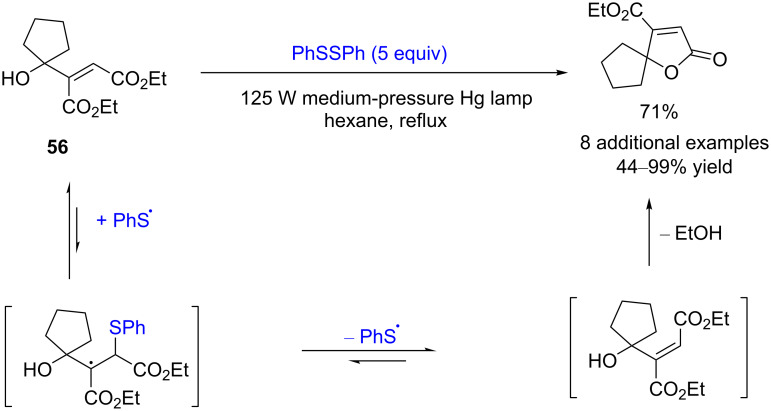
Disulfide-catalyzed conversion of maleate esters to fumarates and 5*H*-furanones.

Thiyl radical-mediated isomerizations of C=C bonds are an effective method in organic synthesis. In 1999, Harrowven and co-workers reported a photoinduced diphenyl disulfide-catalyzed conversion of maleate esters to fumarates and 5*H*-furanones [28]. Five equivalents of diphenyl disulfide and a maleate derivative, such as **56**, were refluxed in hexane for 4–72 hours under irradiation from a 125 W medium-pressure mercury lamp, and 5*H*-furanones were obtained in moderate to good yield (44–77%, Scheme 21). Under the same reaction conditions, unsubstituted maleate esters were also nearly quantitatively converted to the corresponding fumarate esters.

**Scheme 22:**
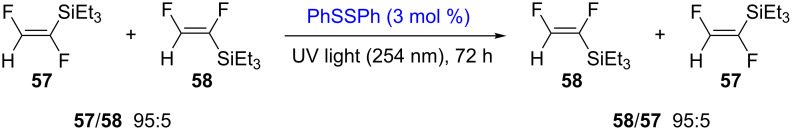
Disulfide-catalyzed isomerization of difluorotriethylsilylethylene.

In 1996, Burton and co-workers reported the stereoselective synthesis of *cis*-1,2-difluorotriethylsilyethylene and its conversion to a variety of *cis*-1,2-difluoroethylene synthons, which are important building blocks in the preparation of fluorine-containing pharmaceuticals, polymers, and bioactive compounds. In the synthesis of these synthons, the key transformation, the isomerization of the *trans*-1,2-difluorotriethylsilylethylene **57** to the *cis* isomer **58**, is realized with phenyl disulfide under ultraviolet-light irradiation (Scheme 22) [29].

**Scheme 23:**
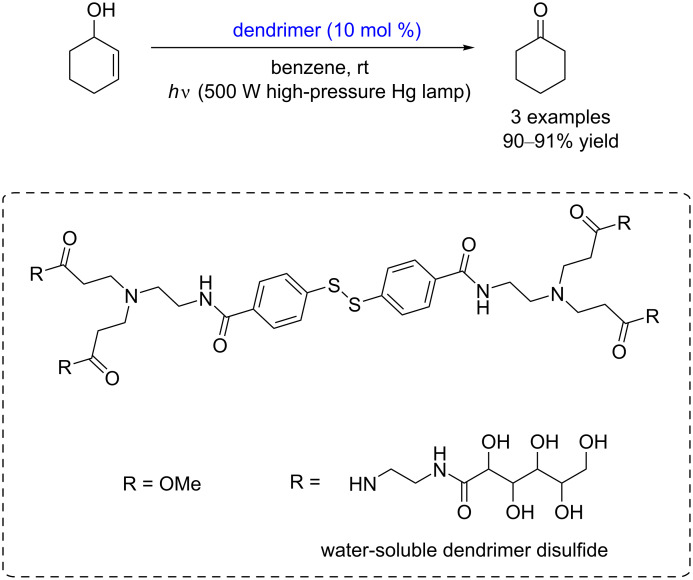
Disulfide-catalyzed isomerization of allyl alcohols to carbonyl compounds.

In 2009, Tsuboi and co-workers also reported a dendrimer disulﬁde-catalyzed isomerization of allyl alcohols to carbonyl compounds under high-pressure mercury lamp irradiation (λ > 300 nm) at room temperature. The yield of the reaction reached up to 91% (Scheme 23) [30]. Using the water-soluble dendrimer disulfide, the photoinduced isomerization in water was also successful. For the photoinduced oxidation reaction of allyl alcohols, the dendrimer disulfide is a better mediator than diphenyl disulfide, because dendritic substituents are quite effective in inhibiting side reactions initiated by the addition of sulfanyl radicals to the unsaturated bonds.

**Scheme 24:**
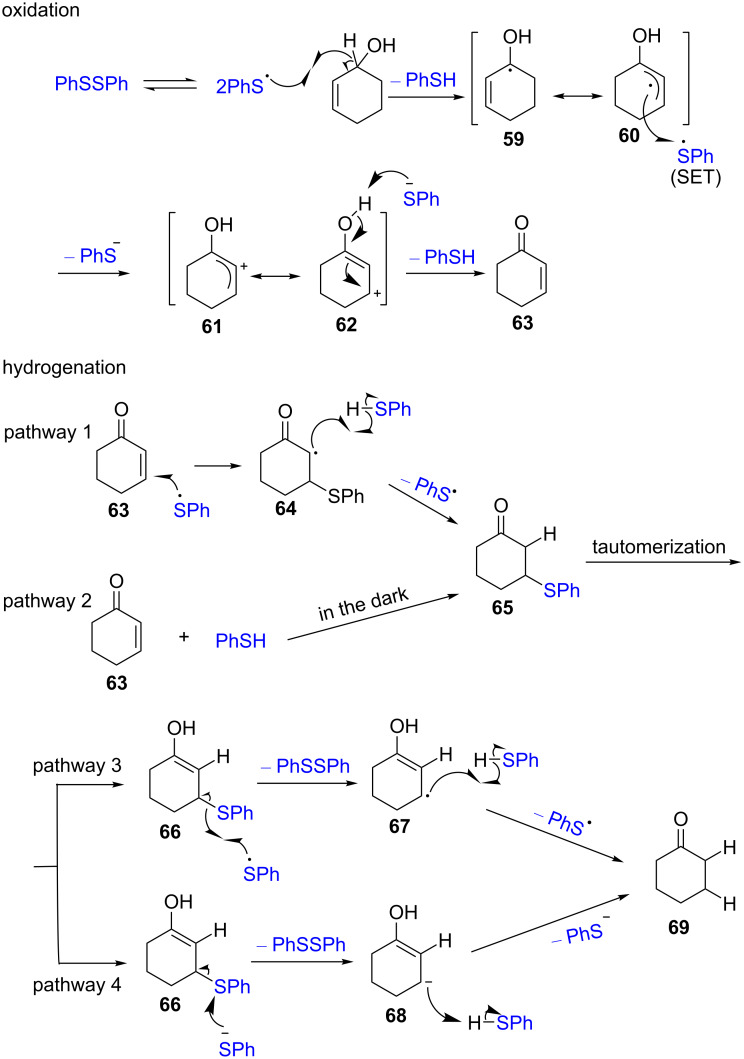
Proposed mechanism for the disulfide-catalyzed isomerization of allyl alcohols to carbonyl compounds.

A plausible mechanism of the catalytic isomerization is shown in Scheme 24. The oxidation step of isomerization is initiated by thiyl radicals, which are generated under illumination. Then, the formed thiyl radical abstracts an α*-*hydrogen atom from allyl alcohols to give thiophenol and the allylic radical **59**. The subsequent SET from the radical **60** to the thiyl radical yields the allylic cation **61** and a thiolate anion. The following proton abstraction by this thiolate anion from the intermediate **62** gives the enone **63** and thiophenol to complete the oxidation. In the hydrogenation step, the addition of a thiyl radical to the enone **63** and posterior hydrogen transfer from thiol to the intermediate **64** forms the ketone intermediate **65** (pathway 1). This ketone intermediate **65** is also accessible by an ionic addition in the dark (pathway 2). The tautomerization of the ketone **65** yields the enol form **66**, which reacts with a thiyl radical (pathway 3) or a thiolate anion (pathway 4) to regenerate the disulfide. Finally, the formed intermediates **67** and **68** furnish the final product **69** by hydrogen (pathway 3) or proton transfer processes (pathway 4), respectively.

**Scheme 25:**
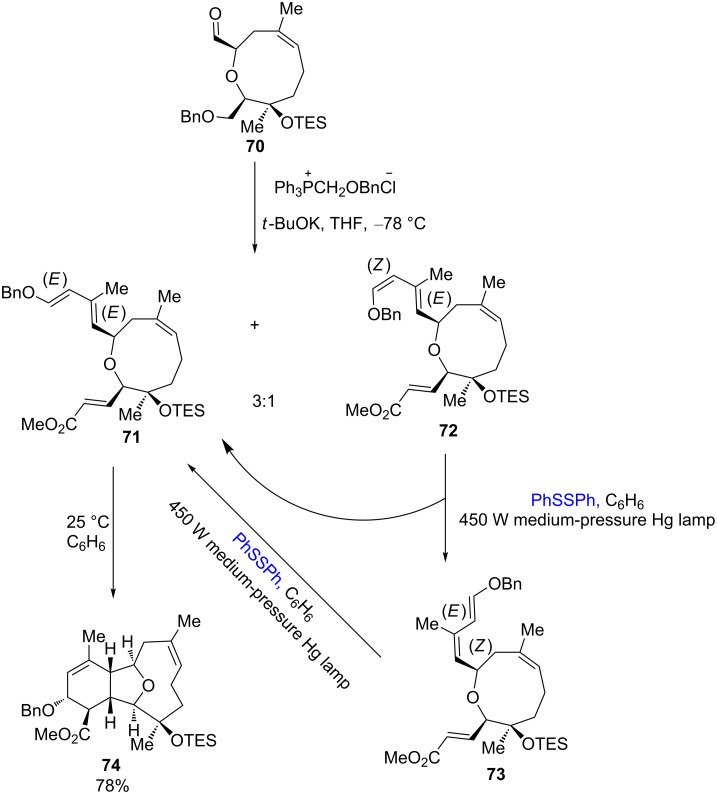
Diphenyl disulfide-catalyzed enantioselective synthesis of ophirin B.

Disulfide-catalyzed isomerization processes also found applications in total synthesis, medium- and large-ring syntheses, and conjugate diene isomerization processes. In 2006, Crimmins successfully used this isomerization method in the syntheses of ophirin B and astrogorgin (Scheme 25) [31]. In the reaction, the intermediate **70** reacts with benzyloxymethylenetriphenylphosphane to give the dienes **71** and **72** as a 3:1 mixture. At room temperature, the resulting (*E*,*E*)-isomer **71** is quantitatively converted into the desired oxatricyclic system **74** via a diastereoselective Diels–Alder cycloaddition process. However, the (*Z*,*E*)-isomer **72** remains unreactive under these reaction conditions. In order to convert the unreactive isomer **72** to the reactive isomer **71**, different isomerization methods were tested. When iodine was used as the isomerization agent, the hydrolysis of the enol moiety took place, and the irradiation with Me_6_Sn_2_ and Bu_6_Sn_2_ did not initiate any isomerization of the (*Z*,*E*)-isomer **72**. Under irradiation with a catalytic amount of diphenyl disulfide, the isomerization of the (*Z*,*E*)-isomer **72** to the (*E*,*E*)-isomer **71** was successfully achieved. In the reaction, the irradiation of the (*Z*,*E*)-isomer **72** with diphenyl disulfide also produced the (*E*,*Z*)-isomer **73**. But in the presence of PhSSPh, the new isomer **73** could also be converted to the desired (*E*,*E*)-isomer **71**.

**Scheme 26:**
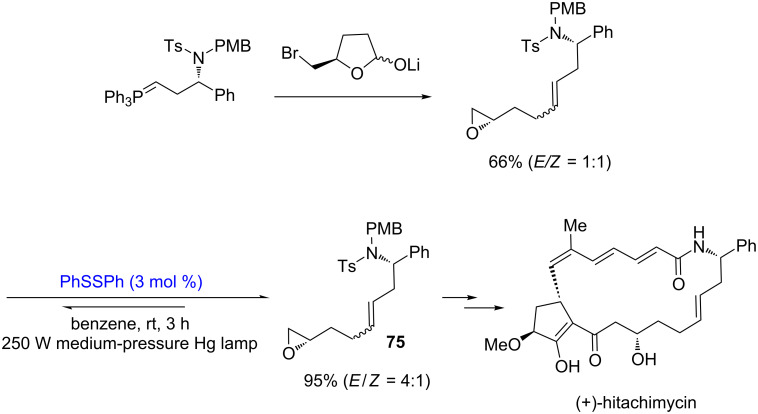
Disulfide-catalyzed isomerization in the total synthesis of (+)-hitachimycin.

In the total synthesis of the macrocyclic antibiotic antitumor agent (+)-hitachimycin, Smith used a disulfide-catalyzed isomerization [32] to synthesize the intermediate **75** with an *E*-configuration, which was unsuccessful to be prepared by the Schlosser modiﬁcation of the Wittig oleﬁnation (Scheme 26) [33].

**Scheme 27:**
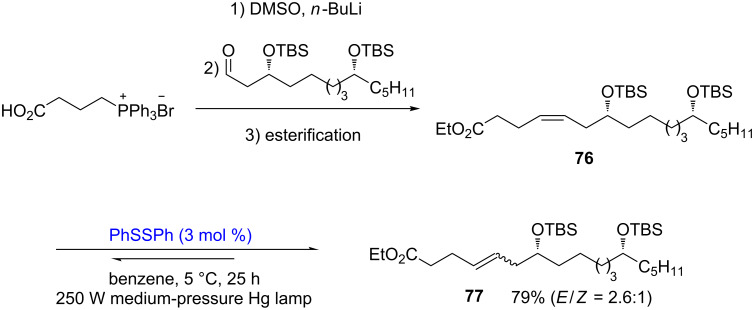
Disulfide-catalyzed isomerization in the synthesis of (−)-gloeosporone.

A similar isomerization method was also used by Holmes in the synthesis of (−)-gloeosporone. Under irradiation conditions, diphenyl disulﬁde helped to convert the *Z*-alkene **76** to the *E*-alkene **77** (Scheme 27) [34].

## Conclusion

In conclusion, under photoirradiation, organic disulfides can be easily cleaved into free radicals and can reversibly add to unsaturated multiple bonds to catalyze a variety of functionalization reactions under mild conditions. Disulfide-catalyzed oxidations of alkenes and alkynes are highly attractive because the reagents involved in the reaction process are simple and inexpensive, plus they only require molecular oxygen or air. In addition, disulfide-catalyzed cyclization reactions are also very effective for the generation of five- and six-membered rings. The unique high selectivity and mildness of disulfide-catalyzed isomerizations enables the broad applications of this method, especially in cases where this cannot be achieved by other isomerization methods. Overall, it should become an effective tool in the organic chemist’s toolbox. Although disulfide-catalyzed photoreactions have gained some interest from researchers, their full potential has not been discovered and released yet. They are environmentally friendly and efficient catalyst that deserve more attention.
